# microRNA-144/451 decreases dendritic cell bioactivity *via* targeting interferon-regulatory factor 5 to limit DSS-induced colitis

**DOI:** 10.3389/fimmu.2022.928593

**Published:** 2022-07-29

**Authors:** Zhijie Lin, Xiaoyan Xie, Min Gu, Qian Chen, Guotao Lu, Xiaoqin Jia, Weiming Xiao, Jun Zhang, Duonan Yu, Weijuan Gong

**Affiliations:** ^1^ Department of Immunology, Institute of Translational Medicine, Medical College, Yangzhou University, Yangzhou, China; ^2^ Jiangsu Key Laboratory of Experimental & Translational Non-coding RNA Research, Yangzhou University, Yangzhou, China; ^3^ Department of Gastroenterology, Affiliated Hospital of Yangzhou University, Yangzhou University, Yangzhou, China; ^4^ Jiangsu Key Laboratory of Integrated Traditional Chinese and Western Medicine for Prevention and Treatment of Senile Diseases, Yangzhou University, Yangzhou, China; ^5^ Department of Blood Transfusion, Affiliated Hospital of Yangzhou University, Yangzhou University, Yangzhou, China; ^6^ Jiangsu Key Laboratory of Zoonosis, Jiangsu Co-innovation Center for Prevention and Control of Important Animal Infectious Diseases and Zoonoses, Yangzhou University, Yangzhou, China

**Keywords:** miR-144/451, dendritic cell, interferon-regulatory factor 5, inflammatory bowel disease, immune regulation

## Abstract

The microRNAs miR-144/451 are highly conserved miRNA that is strongly induced during erythropoiesis. Despite the biological functions of miR-144/451 have been extensively studied in erythropoiesis and tumorigenesis, few studies have been conducted in immune responses. In this study, we showed that miR-144/451^-/-^ DCs exhibit increased activation. Mechanistically, the miR-144 directly targets the 3`-UTR of IRF5 and represses the expression of IRF5 in DCs. Ectopic expression of miR-144/451 by lentiviruses downregulates the levels of IRF5 and suppresses DCs function. In addition, knockdown of IRF5 by shRNA significantly inhibits activities of the miR-144/451^-/-^ DCs. Expression of miR144/451 was decreased in DCs from both patients with IBD and mice with DSS-colitis compared with controls. Human PBMC derived DCs were downregulated expression of miR144/451 after LPS stimulation. In the DSS-induced colitis mice model, we showed that ablation of the miR-144/451 gene causes severe colitis, and their DCs from both periphery and MLN expressed higher co-stimulatory molecules and pro-inflammatory cytokines than wild-type mice. In addition, DCs isolated from miR-144/451^-/-^ mice transfusion exacerbates mice colitis. In the bone marrow transplanted chimeric mice model, we show that miR-144/451^-/-^ bone marrow transplantation deteriorated DSS-induced colitis. At last, we treat the mice with miR-144/451 delivered by chitosan nanoparticles revealing protective effects in DSS-induced colitis mice. Thus, our results reveal a novel miR144/451-IRF5 pathway in DCs that protects experimental colitis. The manipulation of miR-144/451 expression and DCs activation in IBD patients may be a novel therapeutic approach for the treatment of inflammatory diseases.

## Introduction

Inflammatory bowel diseases (IBD), including two main clinical entities: Cohn’s disease (CD) and ulcerative colitis (UC) are characterized by both acute and chronic inflammation in the intestine. The etiology and pathogeny of IBD are largely unknown. Accumulating evidence have revealed that IBD is caused by dysregulated function of local immune cells such as dendritic cells (DCs), macrophages and T cells, and context cytokine production in the intestine (1, 2).

MicroRNAs are endogenous short (~19 to 22 nucleotides) RNA molecules that negatively regulate gene expression in animals and plants by targeting mRNAs for degradation or translation inhibition (3). There is increasing evidence indicating that miRNAs play important regulatory roles in immunity, and then contribute to both physiology and pathophysiology (4–6). Emerging studies from large IBD patient cohorts, together with mouse models that develop chronic intestinal inflammation resembling human IBD, have revealed that miRNAs regulate immune responses and contribute to IBD progression (7–9).

miR-144 and miR-451 are two highly conserved miRNA encoded by a bicistronic gene locus *miR-144/451* that is strongly induced during erythropoiesis and facilitates erythrocyte maturation in zebrafish, mice and human (10–12). GATA1, a master transcription factor that regulates erythropoiesis plays an essential role in miR-144/451 transcription (13). Our previous studies demonstrate that miR-451 directly targets mRNAs of *Cab39*, *Ywhaz* (encoding protein 14-3-3ζ) and *c-Myc* followed by activation of the downstream pathway in erythroid cells and B-lymphocytes (14–16). miR-144 directly targets the nuclear factor erythroid-2-related factor-2 (Nrf2) for mRNA degradation in hepatocellular carcinoma cell (17). A recent study shows that miR-451 limits T cell proliferative responses to infection in mice (18). In addition, we have found that miR-144/451 KO mice show increased antitumor immune responses of CD8^+^T cells to colorectal cancer and melanoma xenograft (Lin et al., unpublished data, 2022). Together, those evidences indicate that miR-144/451 contributes to the regulation of immune responses. However, the molecular mechanisms remain largely unknown.

Interferon regulatory factor (IRF) family are transcription factor comprises nine members in human, which play a critical role in antiviral defense, immune response, cell growth regulation and apoptosis (19, 20). IRF1 and IRF2 are directly bound to the positive regulatory domain 1 (PRDI) of the IFN-β gene to activate and repress the expression of the IFN-β gene. IRF3 and 7 are involved in the signal transduction mediated by virus, which is critical for the transcriptional activation of Type I IFN gene. While IRF-5 plays a role in apoptosis and inflammatory responses to pathogens. IRF5 is constitutively expressed in innate immune cells including (DCs), macrophages, and neutrophils. IRF5 is also induced in adaptive immune cells upon activation of the Toll-like receptor pathway (21, 22). Accumulating studies indicate that multiple polymorphisms of the IRF5 gene are involved in autoimmune diseases, such as rheumatoid arthritis (RA), systemic lupus erythematosus (SLE) (23, 24), and IBD (25, 26).

In the current study, we use miR-144/451 knockout mice, dextran sulfate sodium (DSS)-induced colitis model, and human IBD patient samples to demonstrate that miR-144/451 expression is decreased in DCs from IBD patients and DSS induced colitis compared with that from healthy donors. We also find that miR-144 targets 3`-UTR of IRF5 mRNA to deregulate DCs activation and ability to prime T cells. Elevated activation of DCs from miR-144/451 KO mice acerbates DSS-induced colitis. Thus, our data indicate that miR-144/451 may repress DCs activation in DSS-induced colitis by targeting IRF5.

## Methods

### Animals and cell line

miR-144/451 knockout (KO, C57BL/6J background, CD45.2^+^) mice lacking a 388 base pair segment of genomic DNA containing both the miR-144 and miR-451 precursors have been described previously (14). C57BL/6J wild type (WT, CD45.2^+^) mice were provided by the Comparative Medical Center of Yangzhou University (Yangzhou, China). CD45.1^+^ WT (C57BL/6J background) mice were obtained from the Jackson Laboratory (Bar Harbor, ME, USA). All animals used in this study were 8-12 weeks old. 293T cells from American Type Culture Collection were maintained in Dulbecco’s modified Eagle’s medium (DMEM) supplemented with 10% fetal bovine serum (FBS), streptomycin and penicillin.

### Human samples

Human peripheral blood samples were obtained from affiliated hospital (Yangzhou University, Yangzhou, China). The written informed consent was provided from each individual.

### miR-144/451 overexpression

420 base pairs of miR-144/451 genomic DNA were cloned and inserted into a pLVX-acGFP1 lentiviral expression vector. Lentiviruses were packaged with vector pLP1, pLP2 and pLP/VSVG in 293T cells by Lipofectamine 3000 Reagent (ThermoFisher). 293T cells or DCs were infected with lentiviruses together with polybrene (10 µg/ml) for 24 or 48 hours. The infection rate of lentivirus was tested in 293T cells as shown in **Supplementary Figure 3A**.

### IRF5 knockdown

IRF5-shRNA was designed using BLOCK-iT RNAi Designer (http://rnaidesigner.thermofisher.com/rnaiexpress, ThermoFisher). shRNA1: 5`- GGGACAACACCATCTTCAAGGCTCGAGCCTTGAAGATGGTGTTGTCCCTTTTT-3`, shRNA2: 5`-GGTTGCTGCTGGAGATGTTCTCTCGAGAGAACATCTCCAGCAGCAACCTTTTT-3`. Target sequences and negative control NC-shRNA (provided by GeneCreate, Wuhan, China) were inserted in the pLVx-shRNA2 vector. Lentiviruses were packaged in 293T cells.

### Antibodies and flow cytometry

Antibodies used in flow cytometry include anti-mouse-CD11b (M1/70), CD11c (N418), CD80 (16-10A1), CD86 (GL-1), H2K^b^ (5041.16.1), I-A/I-E (M5/114.15.2), CD1d (1B1), TNF-α (MP6-XT22), IL-6 (MP5-20F3), CD3 (17A2), CD4 (GK1.5), CD8 (53-6.7), CD69 (H1.2F3), NKG2D (CX5), IFN-γ (XMG1.2) and anti-human-CD11b (M1/70), CD11c (3.9), CD80 (2D10.4), CD86 (IT2.2), TNF-α (MAb11) were obtained from BioLegend (San Diego, CA, USA) or Thermo Fisher (Waltham, Mass, USA). Cells were treated or permeabilized, stained with corresponding antibodies, and analyzed by BD FACSVerse system and FlowJo software (version 10.4, FlowJo LLC, Franklin Lakes, NJ, USA).

### Cell sorting

CD11c^+^ DCs (PE anti-CD11c and anti-PE MicroBeads, Miltenyi Biotec), CD8^+^T (Ly-2 MicroBeads), and CD4^+^T (L3T4 MicroBeads) cells were sorted by magnetic activated cell sorting (MACS) according to the manufacturer’s protocol.

### Quantitative real-time PCR (qRT-PCR)

Cells with corresponding treatment were harvested and stored in TRizol reagent (Invitrogen, CA, USA). Total RNA was extracted and assessed quality by NanoDrop (Thermo Fisher). RNA was reverse transcribed to cDNA using Takara Prime Script RT reagent kit with gDNA Eraser (Takara Bio Inc). The primer sequences used in this study were as follow, IRF5: foreword-5`-CCTCCCAACGCACCCTATT-3`, reverse -5`-ATCAGCAGGTCAGGCAAGA-3`. GAPDH: foreword-5`- CCACTCACGGCAAATTCAAC-3`, reverse -5`-CTCCACGACATACTCAGCAC-3`. The relative expression level was calculated using the 2^−ΔΔ^CT method. Primers for microRNA quantification were designed by miRAN Design software (Vazyme Biotech, Nanjing, China): RT-miR-144-5`-GTCGTATCCAGTGCAGGGTCCGAGGTATTCGCACTGGATACGACACTTAC-3`, miR-144F5`-GCGCGGGATATCATCATATACT-3`; RT-miR-451-5`-GTCGTATCCAGTGCA GGGTCCGAGGTATTCGCACTGGATACGACAACTCA-3`; miR-451F-5`-CGCGAAACCGTTACCATTAC -3`. miRNA 1st Strand cDNA Synthesis Kit by stem-loop and miRNA Universal SYBR qPCR Master Mix (Vazyme Biotech) were used for microRNA quantification.

### Western blot

Fresh isolated or treated DCs were rinsed with cold PBS and lysed in lysis buffer (Beyotime Biotechnology, Shanghai, China). The protein concentration was measured using NanoDrop. Equal amounts of protein were separated by 12% SDS-PAGE and transferred to polyvinylidene fluoride membranes; nonspecific sites were blocked with 5% BSA in TBST and the membranes were then incubated with dilutions of the primary antibody as recommended by the manufacturer. The antibodies used are as follows: anti-IRF5(1:1000, 96527), anti-β actin (1:5000, 3700) (CellSignaling, Beverly, MA, USA), HRP conjugated anti-rabbit secondary antibody (1:2000, Invitrogen, Grand Island, NY, USA). Western blots were visualized using the enhanced chemiluminescent (ECL) reagent (Thermo Fisher).

### Dual-luciferase reporter assay

228 base pair segments of IRF5 3’-UTR containing the target sequences (wild type and mutated) of miR-144 were cloned into the pGL3-BS vector (Supplementary **Figure 2D**) (14). 293T cells were transfected with the pGL3-BS plasmid using the Lipofectamine 3000 transfection reagent (Invitrogen). The pRL-TK vector was used as an internal control reporter. The luciferase activities were monitored 24 hours post-transfection using the Promega Dual-Luciferase Reporter assay system (Promega Corporation, Madison, Wisconsin, USA).

### DSS-induced colitis

8-12 weeks old female mice were fed with DSS (2.5%, molecular weight 36000–50000, MP Biomedicals, Santa Ana, CA, USA) in drinking water for 6 days followed by DSS-free water (27). The body weight changes, diarrhea, and stool conditions were documented daily and used to assess the disease activity of colitis. The disease activity index (DAI) was measured as reported previously.

### Bone marrow and DC transplantation

In the BMT model, recipient mice were pretreated with busulfan (i.p. three times of 90 mg/kg total dose per mouse. *i.e.*, for 20 g mouse, administer 600 µg of busulfan at day -7, -5 and -3). Donor bone marrow cells were freshly isolation from femur and tibia of donor mice, and then adoptively transferred to recipient mice (2 × 10^6^ bone marrow cells per mouse) *via* tail vein. Recipient mice were supplied with water containing gentamicin and erythromycin for 2 weeks before the followed procedures. For the DCs transplantation model, CD11c^+^ DCs were sorted and transferred to recipient mice (2 × 10^6^ cells/mouse *via* tail vein).

### H&E stain

Mouse colonic tissues were fixed and embedded in paraffin according to standard procedures. Colon sections (5 μm) were mounted on glass slides and stained with hematoxylin and eosin (H&E). Pathological evaluations were performed based on an earlier study (28).

### Preparation of chitosan-plasmid nanoparticles

Chitosan nanoparticles were prepared as previously described (29). The solution of the control vector or vector containing pri-miR-144/451 segments (1 mg/ml) was dropwisely added into an equal amount of chitosan solutions (1 mg/ml). Mixed solutions were shaken and centrifuged, the sediments were dissolved with PBS (1 mg/ml). For *in vivo* delivery study, mice were treated with nanoparticles daily (chitosan-plasmid, 100 μg/mouse).

### Statistical analysis

All data were presented as the means ± SEM. Differences between two samples were analyzed using an unpaired, two-sided Student’s t-test. Multiple comparisons were performed using two-way ANOVA, and p values were adjusted using Tukey’s method. p values of less than or equal to 0.05 were considered statistically significant. Statistical analyses were performed using GraphPad Software Prism 9. Statistical significance was indicated as *p<0.05, **p<0.01, ***p<0.001.

## Results

### MiR-144/451 ablation upregulates DC activation

**Figure 1:**
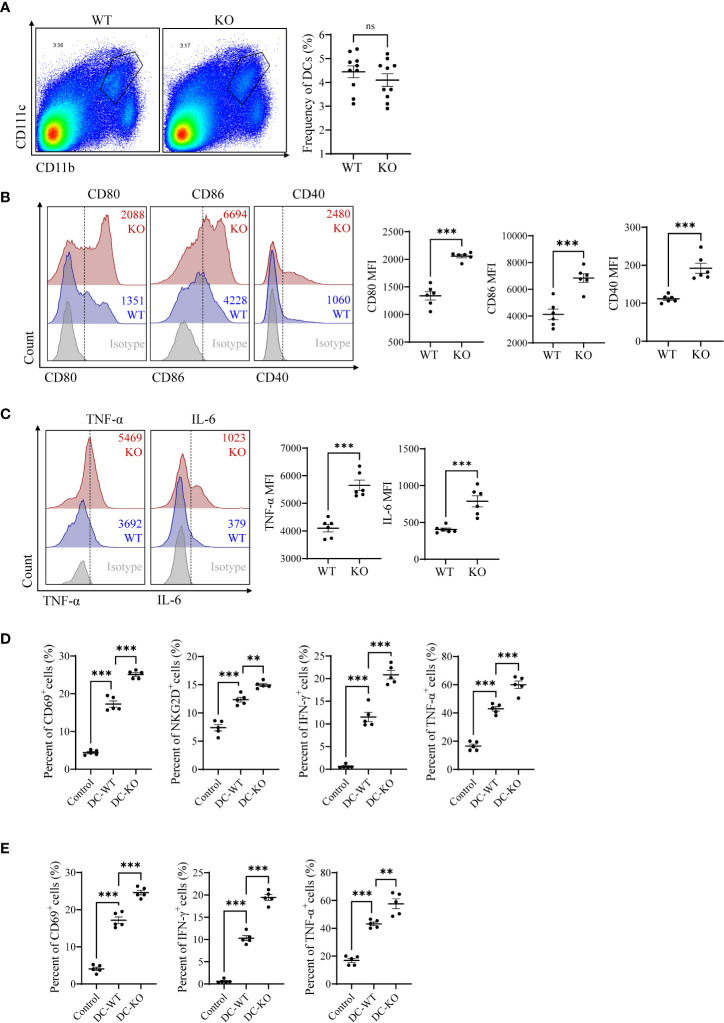
miR-144/451 ablation upregulates DCs activation. Frequency of CD11b^+^CD11c^+^ DCs in the spleen of WT or miR-144/451 KO mice **(A)**. Expression of CD80, CD86 and CD40 on splenic DCs **(B)**. Intracellular staining of TNF-α and IL-6 on splenic DCs stimulated 5 h with PMA/Ionomycin plus BFA **(C)**. CD8^+^T cells **(D)** and CD4^+^T cells **(E)** were co-cultured with WT or miR-144/451 KO DCs for 24 h, expression of surface markers CD69, NKG2D and intracellular cytokines IFN-γ, TNF-α was detected. Ns. no significance, **p<0.01, ***p<0.001.

We first evaluated the DCs activities in miR-144/451 KO mice under physiological conditions. The frequency of DCs was similar between KO and WT mice (**Figure 1A** and **Supplementary Figure 1A**). Whereas the expression of costimulatory molecules (CD80, CD86 and CD40) were significantly upregulated in DCs from KO mice compared to those with WT mice (**Figure 1B** and **Supplementary Figure 1B**). The expression of antigen presentation molecules (H2K^b^, I-A/I-E, CD1d) was not dramatically changed in DCs from KO mice (**Supplementary Figures 1C, D**). Simultaneously, elevated levels of pro-inflammatory factor TNF-α and IL-6 were observed in DCs from KO mice (**Figure 1C**). DCs are the main antigen-presenting cells for T cell activation and polarization. We therefore determined the ability to prime T cells of DCs in co-culture system. DCs from both WT and KO mice shows promoted T cell activation after co-culture. Notably, the DCs from KO mice showed the potent capacity for T priming than DCs from WT mice (**Figures 1D**, **E** and **Supplementary Figures 1E–G**). Together, these findings indicated that miR-144/451 inhibits DC activation in mice.

### Identification of IRF5 as a target of miR-144/451

**Figure 2:**
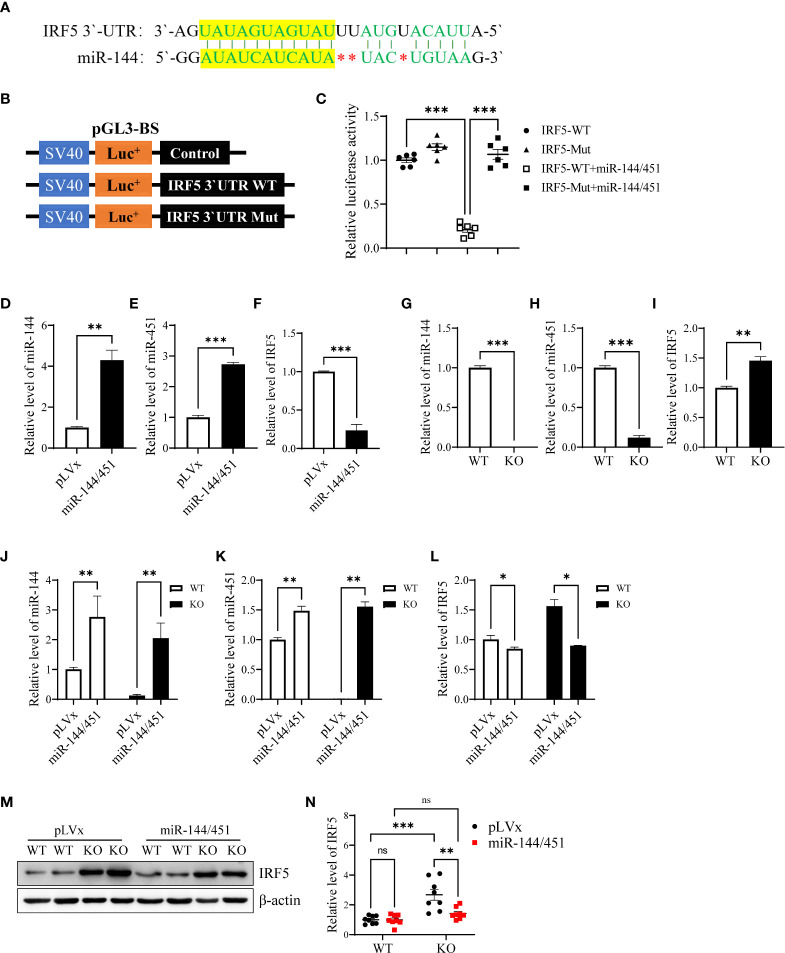
Identification of IRF5 as a target of miR-144/451. Nucleotide sequence alignments showing complementarity between the 3′-UTRs of IRF5 mRNAs and miR-144 **(A)**. Native and mutant version 3′-UTRs of IRF5 mRNAs were inserted into the pGL3-BS vector **(B)**, and statistic bars represent the Firefly/Renilla luciferase activity detected in 293T cells **(C)**. Expression of miR-144 **(D)**, miR-451 **(E)** and IRF-5 Mrna **(F)** was detected in 293T cells after 24 h lentiviruses infection. Expression of miR-144 **(G)**, miR-451 **(H)** and IRF-5 mRNA **(I)** were detected in fresh isolated DCs from the spleen of WT or miR-144/451 KO mice. Expression of miR-144 **(J)**, miR-451 **(K)** and IRF-5 mRNA **(L)** were detected in DCs 24 h after lentiviruses infection (n = 3). Expression of IRF-5 protein was assayed in DCs 48 h after lentiviruses infection **(M)**, and bars represent the ratio of IRF-5/β-actin **(N)**. Ns. no significance, *p<0.05, **p<0.01, ***p<0.001.

To investigate whether miR-144/451 directly represses DCs function by regulating genes expression. We performed transcriptome sequencing. In silico analyses, using the KEGG cluster profile, we found that the inflammatory pathways including chemokine, NF-κB, TLR signaling pathways were significantly changed (**Supplementary Figure 2A**). Meanwhile, we searched several online databases, the sequence of miR-144 is partially complementary to a sequence within the 3′-UTRs IRF5 mRNA (**Figure 2A**). Previous study reveals that IRF-5 critically contributes to TLR mediated production of pro-inflammatory cytokine TNF-α and IL-6 in DCs (30). Up-regulation of IRF5 transcription was also observed in KEGG pathway and heat map analyses (**Supplementary Figures 2B, C**). We therefore constructed luciferase reporter plasmid pGL3 containing native or mutant version 3′-UTRs of IRF5 mRNA (**Figure 2B** and **Supplementary Figure 2D**). In luciferase reporter assay, luciferase activity in cells with native 3′-UTRs of IRF5 mRNA was dramatically inhibited by co-transfection with miR144/451. Mutation of IRF5 mRNA 3′-UTRs abrogated repression of luciferase activity (**Figure 2C**).

Next, we overexpressed miR-144/451 in 293T cells using miR-144/451 lentiviruses (**Supplementary Figure 3A**). Expression of miR-144 and miR-451 was increased after miR-144/451 lentiviruses infection (**Figures 2D, E**). In contrast, the expression of IRF5 mRNA was significantly repressed after miR-144/451 lentiviruses infection (**Figure 2F**). Expression of miR-144 and miR-451 was eliminated in DCs from miR-144/451 KO mice (**Figures 2G, H**), whereas the expression of IRF5 was increased in miR-144/451 KO DCs (**Figure 2I**). Overexpression of miR-144/451 in DCs significantly downregulated IRF5 mRNA (**Figures 2J–L**) and protein levels (**Figures 2M, N**). Together, these results verify that miR-144/451 directly represses the expression of IRF5 in DCs.

### Ectopic expression of miR-144/451 downregulates DCs function

**Figure 3:**
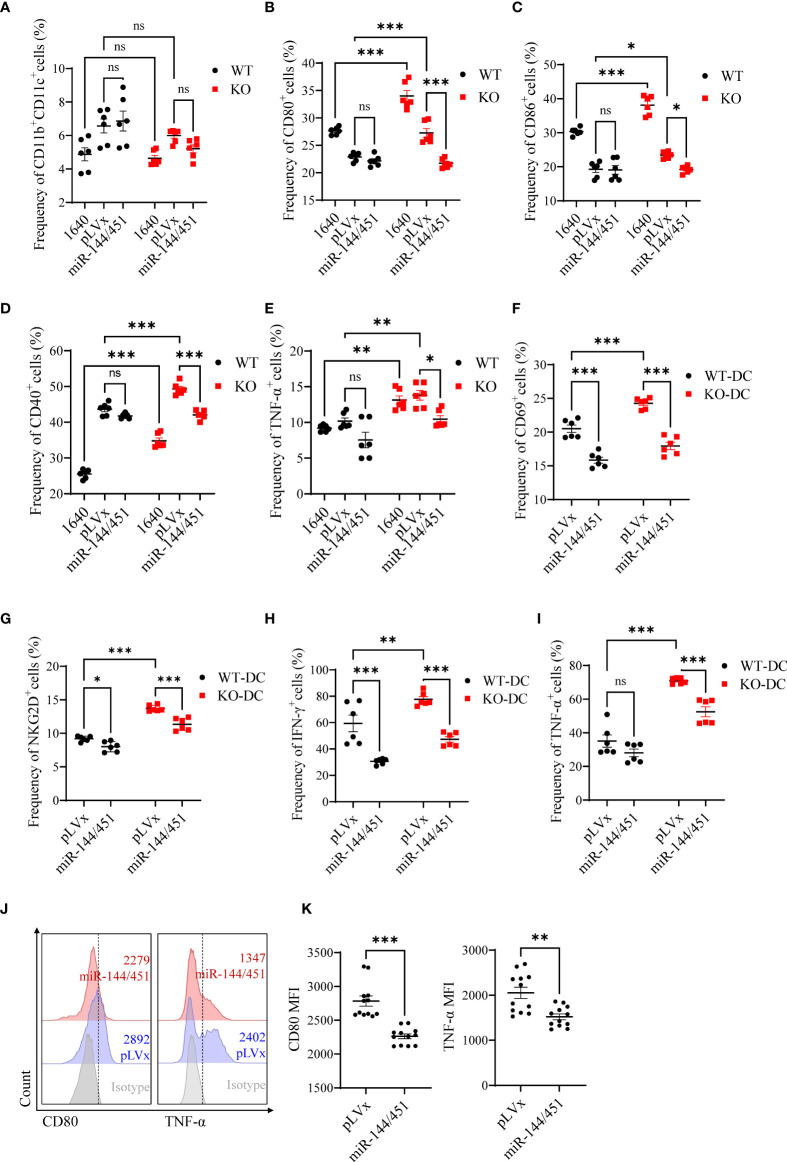
Ectopic expression of miR-144/451 downregulates DCs function. Mice splenic DCs were infected with lentiviruses for 24 h, the frequency of DCs **(A)** and expression of CD80 **(B)**, CD86 **(C)**, CD40 **(D)** and TNF-α **(E)** on DCs was detected. CD8^+^T cells were co-cultured with lentiviruses infected DCs for 24 h, surface markers CD69 **(F)**, NKG2D **(G)** and intracellular cytokines IFN-γ **(H)**, TNF-α **(I)** were detected in CD8^+^T cells. Human PBMCs were cultured in presence of GM-CSF (50 ng/ml) and IL-4 (50 ng/ml) for 5 days. Expression of CD80 and TNF-α **(J, K)** by monocyte-derived DCs were detected after 48 h lentiviruses infection. Ns. no significance, *p<0.05, **p<0.01, ***p<0.001.

To confirm there was a negative correlation between the levels of miR-144/451 and DCs activation. We packed miR-144/451-expressing lentiviruses, and the efficiency of infection was assayed in 293T cells (**Supplementary Figure 3A**). The frequency of CD11b^+^CD11c^+^ splenic DCs was not changed after miR-144/451 ectopic expression (**Figure 3A** and **Supplementary Figure 3B**). Whereas, expression of costimulatory molecules (**Figures 3B–D** and **Supplementary Figure 3C–E**) and TNF-α (**Figure 3E**) on miR-144/451 KO DCs were significantly decreased after ectopic miR-144/451 expression. In addition, the expression of activation molecules CD69, NKG2D (**Figures 3F, G** and **Supplementary Figure 3F**), and cytokine IFN-γ and TNF-α (**Figures 3H, I** and **Supplementary Figure 3G**) in CD8^+^T cells were significantly decreased after co-culture with miR-144/451-overexpressing DCs. Next, we determined whether miR-144/451 overexpression deregulates the activation of human DCs. Human PBMC-derived DCs (**Supplementary Figure 3H**) were isolated and infected with miR-144/451 lentiviruses. The expression of CD80 and TNF-a, but not CD86, were significantly down-regulated in the miR-144/451 group compared with those in control DCs (**Figures 3J, K** and **Supplementary Figure 3I**). Together, these data verify that miR-144/451 directly suppresses the activation of DCs.

### Knockdown of IRF5 inhibited activities of miR-144/451^-/-^ DCs

**Figure 4:**
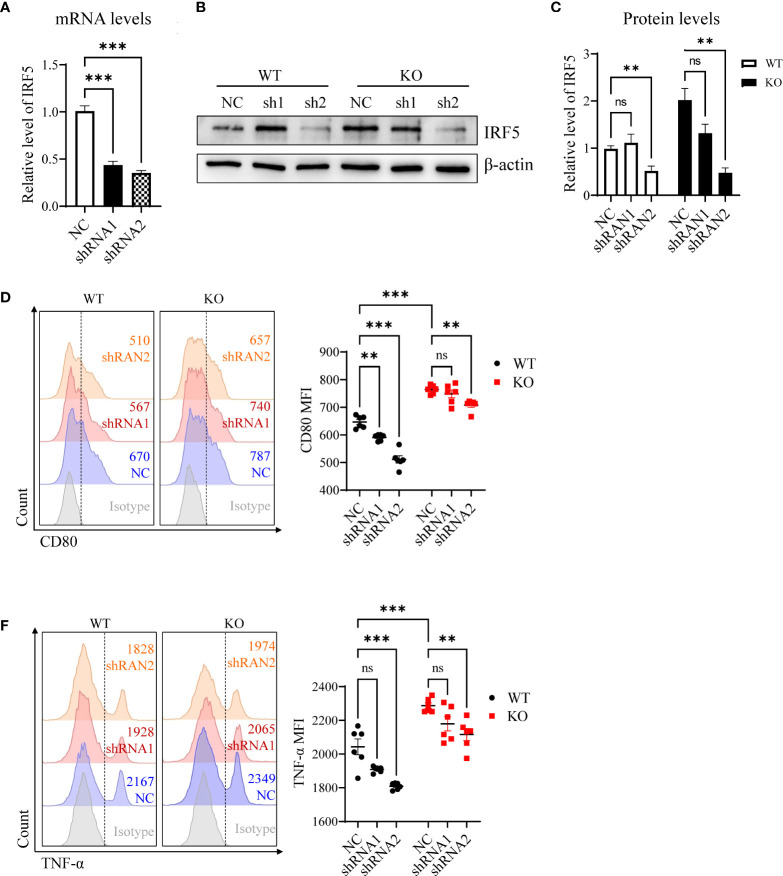
Knockdown of IRF-5 inhibits activities of DCs. Expression of IRF-5 mRNA was detected in DCs after 24 h lentiviruses infection **(A)**. IRF-5 protein level was measured in DCs after 48 h lentiviruses infection **(B)**, and statistic bars represent the ratio of IRF-5/β-actin **(C)** n = 5). Expression of CD80 **(D, E)** and TNF-α **(F, G)** on splenic DCs after lentiviruses infection. The statistical graph represents the mean fluorescence intensity gated on CD11b^+^CD11c^+^ cells. Ns. no significance, **p<0.01, ***p<0.001.

To determine whether miR-144/451 represses DCs function *via* IRF5, we packaged shRNA-expressing lentiviruses to knock down IRF5. We infected DCs with shRNA lentiviruses for 24 h or 48h, then quantitated the IRF5 mRNA expression (**Figure 4A**) and IRF5 protein levels (**Figures 4B**, **C**) in the cells. Knockdown IRF5 in DCs significantly repressed costimulatory molecules CD80 expression (**Figures 4D**, **E**), but no changes in CD86 levels (**Supplementary Figure 4A**). At the same time, TNF-α production was significantly down-regulated in DCs after shRNA2 lentivirus infection (**Figures 4F**, **G** and **Supplementary Figure 4B**). Together, these findings further confirmed that miR-144/451 directly inhibits the expression of IRF5 and that this interaction represses DCs function.

### miR-144/451 KO exacerbates DSS-induced colitis by enhanced DC activation

Dendritic cells are central to the regulation of immune function in the intestine. To investigate whether miR-144/451 abnormality-induced DCs activation is involved in DSS-induced mouse colitis, we first assayed the expression of miR-144/451 and IRF5 in DCs from colitis mice. As shown in **Supplementary Figure 5A**, expression of miR-144/451 was decreased in DCs at day 3, day 5 after mice were treated with DSS, together with significantly upregulated levels of IRF5 (**Supplementary Figure 5A**).

Next, we determined the levels of miR-144/451 and IRF5 in DCs from IBD patients. Similar to colitis mice, miR-144/451 expression was ablated, paralleled with increased IRF5 levels in DCs from IBD (**Supplementary Figure 5B**). If miR-144/451 has negative correlation with DCs activation, decreased expression of miR-144/451 should be observed in DCs under activated conditions. We therefore treated human DCs with LPS to activation, and then assayed the levels of miR-144/451 and IRF5. As expected, repressed miR-144/451 expression and increased IRF5 levels were obtained in LPS treated human DCs (**Supplementary Figure 5C**).

**Figure 5:**
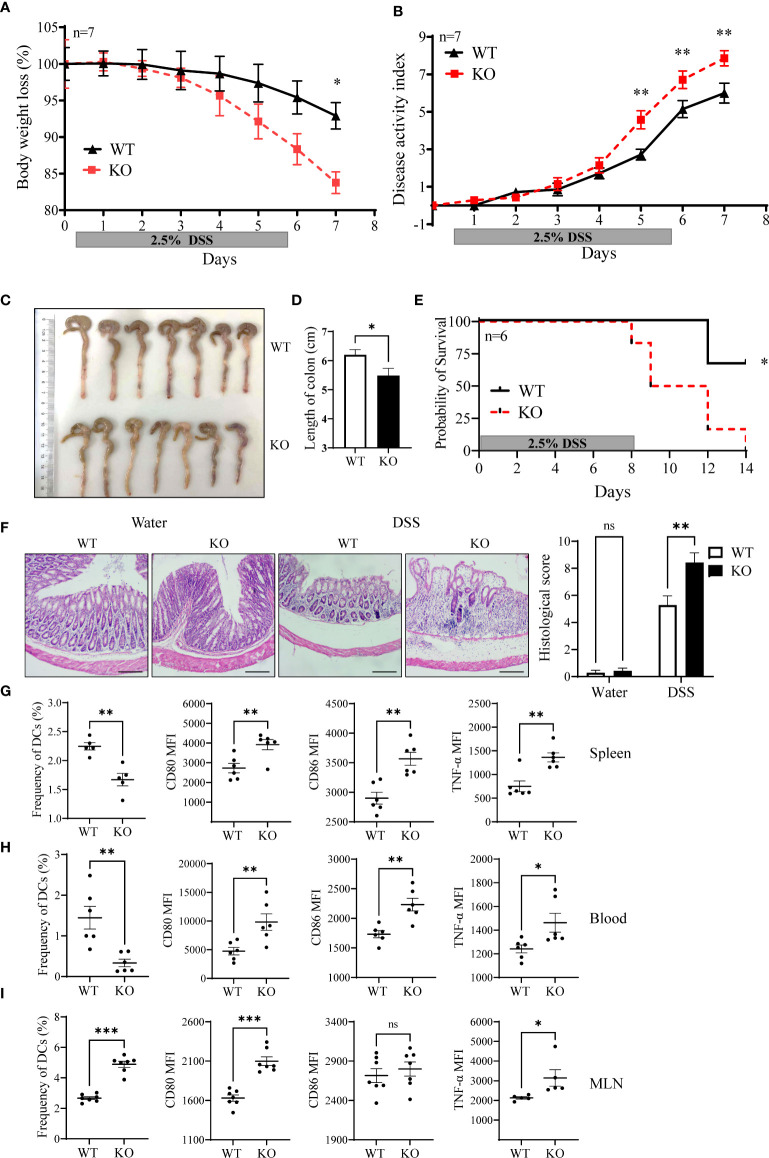
miR-144/451 KO exacerbates DSS-induced mice colitis. WT and miR-144/451 KO mice were treated with 2.5% DSS. Changes in body weight **(A)**. Disease activity index of colitis **(B)**. Morphology of colons **(C)**, and bars represent the length of colons **(D)**. Survival curve of mice with colitis **(E)**. Histological sections and pathological scores **(F)**, 40 ×). Frequency of DCs, and expression of CD80, CD86 and TNF-α on DCs from spleen **(G)**, Blood **(H)** and MLN **(I)** of colitis mice. Ns. no significance, *p<0.05, **p<0.01, ***p<0.001.

We then evaluated the sensitivity of miR-144/451 KO mice to DSS-induced colitis. miR-144/451 KO mice developed severer colitis with a dramatic decrease in body weight, colon length, survivals and increase of disease activity index and pathological scores than WT mice (**Figures 5A–F** and **Supplementary Figure 5D, E**). Additionally, the frequency of DC from the spleen and blood were both decreased in KO mice after DSS treatment (**Figures 5G**, **H**). In contrast, the frequency of DCs was increased in mesenteric lymph nodes of DSS-treated KO mice (**Figure 5I**). Consistent with miR-144/451 KO DC activation in physiological status, expression of CD80, CD86 and TNF-α were increased in DCs of the spleen, blood and MLN from DSS treated KO mice (**Figures 5G–I** and **Supplementary Figure 5F–H**).

**Figure 6:**
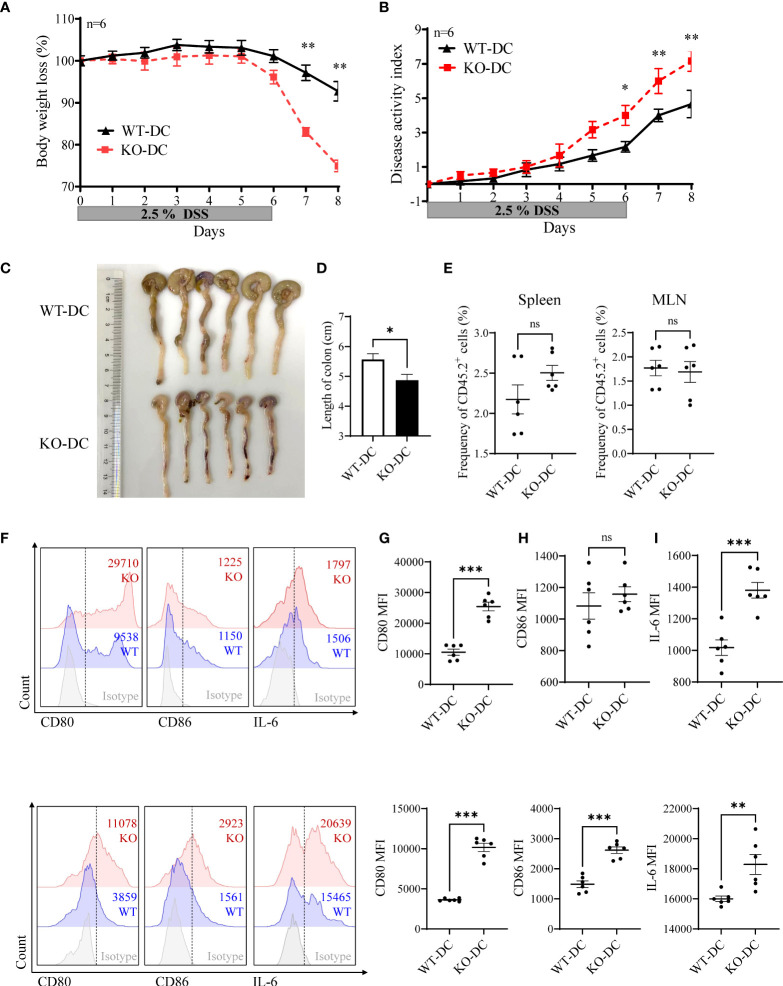
miR-144/451^-/-^ DCs-transplantation exacerbates DSS-induced mice colitis. CD45.1^+^ WT recipient mouse was treated with 2.5% DSS, and adoptively transferred with WT (CD45.2^+^, n=6) or miR-144/451 KO (CD45.2^+^, n=6) DCs *via* tail vein at day -1, day 3 and day 5. Changes in body weight **(A)**. Disease activity index of colitis **(B)**. Morphology of colons **(C)**, and bars represent the length of colons **(D)**. Frequency of CD45.2^+^ cells in spleen and MLN of recipient mice **(E)**. Expression of CD80, CD86 and IL-6 on CD45.2^+^ DCs from spleen **(F, G)** and MLN **(H, I)** of colitis mice. Ns. no significance, *p<0.05, **p<0.01, ***p<0.001.

Next, we sorted the DCs from WT and KO mice, and adoptively transferred them *via* tail vein to DSS treated CD45.1^+^ WT mice. miR-144/451 KO DC transplantation significantly exacerbated DSS-induced colitis, as demonstrated by body weight loss (**Figure 6A**), disease activity index (**Figure 6B**) and colon length (**Figures 6C**, **D**). The frequency of CD45.2^+^ DCs from spleen or MLN was not changed between WT and KO mice (**Figure 6E** and **Supplementary Figure 6A**). Whereas, levels of CD80, CD86 and IL-6 on DCs were increased in miR-144/451 KO DC-transplanted mice (**Figures 6F**– **I**). Together, these data demonstrate that miR-144/451 KO exacerbates DSS-induced colitis through enhanced DCs activation.

### Verification of enhanced DC activation in miR-144/451 KO mice by bone marrow transplantation

**Figure 7:**
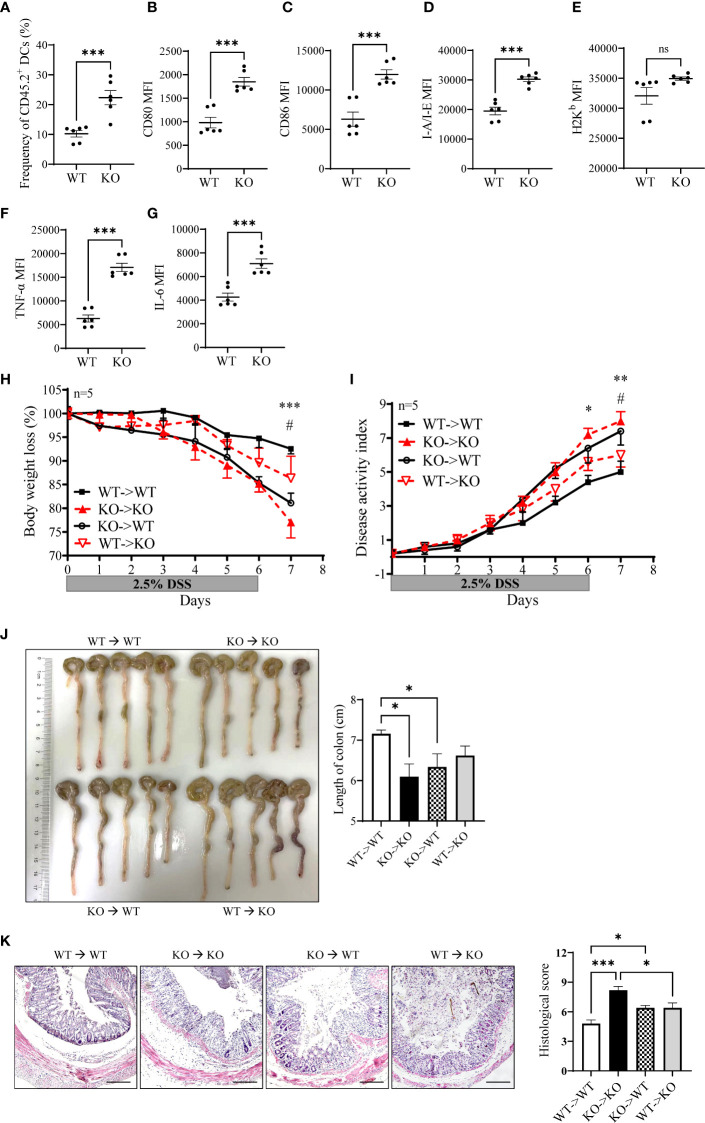
Enhanced DC activation verified by bone marrow transplantation. CD45.1^+^ WT recipient mouse was treated with busulfan and adoptively transferred with bone marrow (CD45.2^+^). Frequency of CD45.2^+^ DCs in the spleen of the recipient **(A)**. Expression of CD80 **(B)**, CD86 **(C)**, I-A/I-E **(D)**, H2K^b^ **(E)**, TNF-a **(F)** and IL-6 **(G)** on CD45.2^+^ DCs. The sensibility of DSS-induced colitis was evaluated in the bone marrow transplantation model. WT (CD45.1^+^) or miR-144/451 KO bone marrow (CD45.2^+^) were injected into busulfan-treated KO or WT mice respectively (n = 5). Changes in body weight **(H)**. Disease activity index of colitis **(I)**. Morphology of colons, and bars represent the length of colons **(J)**. Histological sections and pathological scores **(K)**, 40 ×). *WT to WT vs. KO to KO, ^#^WT to WT vs. KO to WT. Ns. no significance, *p<0.05, **p<0.01, ***p<0.001.

To determine whether increased activation of miR-144/451 KO DCs is due to endogenic regulation or cell development microenvironment. Chimeric mice were established after bone marrow transplantation. CD45.1^+^ WT mice were treated with busulfan (**Supplementary Figure 6B**) and adoptively transferred with WT or miR-144/451 KO bone marrows (CD45.2^+^, **Supplementary Figure 6C**). The frequency of CD45.2^+^ DCs was significantly increased in KO bone marrow-transplanted mice (**Figure 7A** and **Supplementary Figure 6C**). Furthermore, the expression of costimulatory molecules (**Figures 7B, C** and **Supplementary Figure 6D**), antigen presentation molecules (**Figures 7D**, **E** and **Supplementary Figure 6E**) and cytokines (**Figures 7F**, **G** and **Supplementary Figure 6F**) on these DCs were upregulated.

Next, we assayed the sensitivity of chimeric mice to DSS-induced colitis. The bone marrow from CD45.2^+^ miR-144/451 KO mice or CD45.1^+^ WT mice were transplanted to busulfan-treated WT or KO mice respectively (**Supplementary Figure 6G**). In a previous study, we found splenomegaly in miR-144/451 KO mice due to compensatory extramedullary hematopoiesis. After WT bone marrow transplantation, splenomegaly of the KO mice was abrogated (**Supplementary Figure 6H**). Severe colitis in the KO mice was alleviated after WT bone marrow transplantation (**Figures 7H–K**). In contrast, miR-144/451 KO bone marrow transplantation exacerbates colitis in WT mice (**Figures 7H–K**). Together, these results verified that miR-144/451 KO potentiates the deteriorated DSS-induced colitis by DC activation.

### Nanoparticles delivery of miR-144/451 ameliorates DSS-induced colitis in KO mice

**Figure 8:**
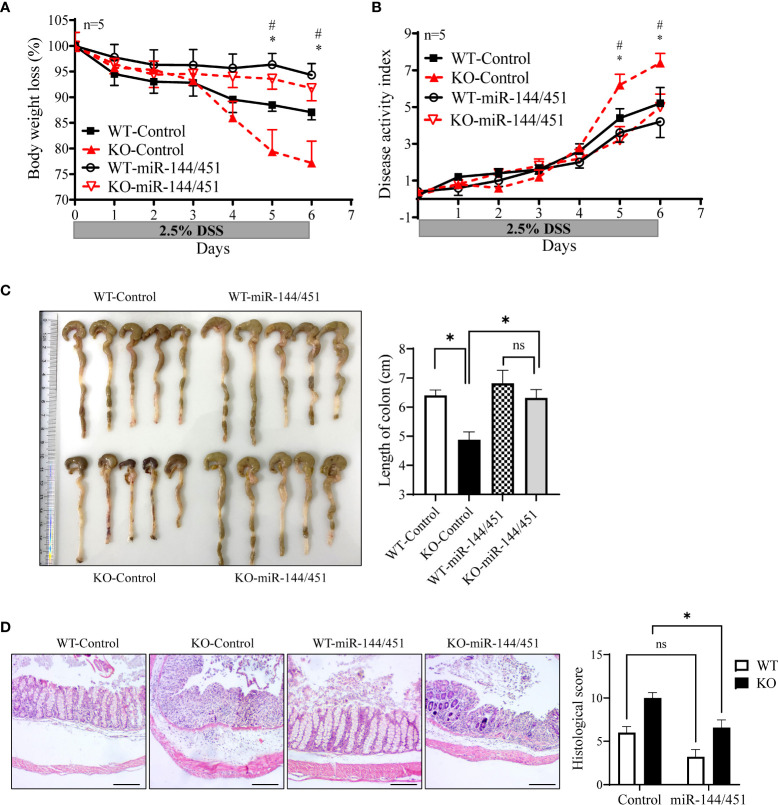
Nanoparticles delivery of miR-144/451 ameliorates DSS-induced colitis in KO mice. WT and miR-144/451 KO mice were treated with 2.5% DSS and intraperitoneally injected with chitosan-plasmid nanoparticles daily. Changes in body weight **(A)**. Disease activity index of colitis **(B)**. Morphology of colons, and bars represent the length of colons **(C)**. Histological sections (40 ×)and pathological scores **(D)**. *WT-Control vs. WT-miR-144/451, ^#^KO-Control vs. KO-miR-144/451, n = 5. Ns. no significance, *p<0.05.

To further confirm the effect of miR-144/451 on DSS-induced mouse colitis. The chitosan nanoparticle-enveloped control vector or miR-14/451 vector were injected intraperitoneally to mice treated with DSS daily. Expression of miR-144, miR-451 and IRF5 were evaluated in DCs of chitosan-miR-144/451 nanoparticle-injected mice (**Supplementary Figures 7A–C**). Compared with the control group, chitosan-miR-144/451 nanoparticle injection alleviated colitis both in WT and KO mice, as demonstrated in less body weight loss (**Figure 8A**), decreased disease activity index (**Figure 8B**), increased colon length (**Figure 8C**) and alleviated histologic damage (**Figure 8D**).

## Discussion

IBD including two major subtypes of clinical entities: CD and UC, are chronic inflammatory diseases of the intestines, and have been shown to multifactorial etiology including dysregulated local immune cell function (1, 2). Innate immune cells, such as macrophages and DCs can sense invading bacteria through extracellular and intracellular pattern recognition receptors such as TLRs and NLRs, and initiate rapid inflammatory responses mediated by the secretion of cytokines and chemokines and recruitment of inflammatory cells (31). The DSS-induced colitis mouse models that develop chronic intestinal inflammation resembling human IBD have been established (27). In the current study, we demonstrated that DCs expression of miR-144 and -451 were significantly decreased in response to LPS and as well as colitis mice. Using the miR-144/451 KO mice to establish DSS-induced experimental colitis model, we found that miR144/451 protect mice intestinal inflammation through repressing TLRs signaling pathway in DCs. We also identified that IRF5 is a direct target gene of miR-144 in facilitating the activation of DCs in colitis.

miRNAs negatively regulate gene expression in a variety of biological processes, and have been implicated in neurological diseases, cardiovascular diseases, cancer and autoimmune diseases (7). Several miRNAs have been suggested in regulating intestinal homeostasis. miR-21 is highly expressed in patients of IBD and experimental colitis models. miR-21 KO mice protecting DSS-induced colitis *via* repress proinflammatory cytokines production and regulates the composition of the intestinal microbiota (32, 33). miR-31 transcription activated in colorectal cancer cells in response to TNF-α and IL-6. miR-31 reduces the inflammatory response in DSS- and TNBS-induced mice colitis by repressing expression of inflammatory cytokine receptors IL7R and IL17RA (9). The key miRNAs involved in IBD, such as miR-21, are the focus of anti-miRNA therapeutic development. In this study, we demonstrated that expression of miR-144/451 was increased in IBD patients. Furthermore, the miR-144/451 KO mice revealed acerbate DSS-induced colitis, which indicated a protective role of miR-144/451 in development of intestinal inflammatory.

miR-144 have being significantly destroyed in many types of cancers including leukemia, gastrointestinal cancers, pancreatic cancer, hepatocellular carcinoma, lung cancer, and breast cancer (34, 35). miR-144 significantly inhibits cell proliferation, metastasis, invasion, EMT, and resistance to chemotherapy of cancer *via* direct targeting genes including B-cell lymphoma 6 (BCL6) (36), human formin-2 (FMN2) (37), nuclear factor erythroid 2–related factor 2 (Nrf2) (17), Smad1 (38), taurine upregulated gene 1 (TUG1) (39) and FMS-like tyrosine kinase 3 (FLT3) (40). Despite the extensive studies of the biological functions of miR-144/451 in erythropoiesis (41) and tumorigenesis (34, 35), few studies have been conducted in immune responses. Our study revealed that DCs activation (this study) and CD8^+^T cell’s anti-tumor responses (Lin et al., unpublished data, 2022) were both elevated in miR-144/451 KO mice. Whereas, we did not see any changes in macrophages including frequency, expression of co-stimulatory factor and MHC molecular in miR-144/451 KO mice compared with that in WT mice (data not show).

Our results identified IRF5 as a target gene of miR-144 to regulate DCs function. Interferon regulatory factor family has been shown to include nine members, IRF1-9 (42). IRF5 is important transcription factor defining the classical inflammatory, which highly expressed in the innate immune cells. Pattern Recognition Receptors (PRRs), such as NOD and TLR regulates activation of IRF5 by phosphorylation and ubiquitination manner. Expression of IRF5 also directly regulates many cytokines including TNF-α, IL-6, IL-1β, which are associated with inflammation responses in central nervous system (43) and intestine (25, 26). Recently, pro-inflammatory role of IRF5 has been suggested in intestinal inflammation. IRF5 promotes inflammatory macrophage polarization and guides monocytes toward and inflammatory CD11c^+^ cells during intestinal inflammation (22, 26). In addition, IRF5 in CD4^+^ T cells promotes Th1 and Th17 associated cytokines, decreases Th2 associated cytokines and enhances the severity of experimental colitis in mice (44). In this study, we found that IRF5 was significantly increased in DCs of patients with IBD and mice with DSS treatment. miR144 targets 3`UTR of IRF5 to suppressing DCs activation and ameliorating DSS-induced colitis in mice. These data thereby indicate that miR-144/IRF5 may be a key regulator in intestinal homeostasis by repressing immune response. However, it is not clear whether miR-451 was involved in this process. Since the miR-451^-/-^ mice had increased responses of T cells to infection (18), in our future studies, it will be interesting to investigate the effects of miR-144 and miR-451 on immune cells activities separately.

Taken together, these findings demonstrated that miR-144/451 regulates DCs function *via* directly targeting IRF5 and which is involved in intestinal inflammation. To our best knowledge, this study was the first to identify miR-144 direct targets 3`-UTR of IRF5 mRNA to deregulate DCs activation. The manipulation of miR-144/451 expression and DCs activation in IBD patients may be a novel therapeutic approach for the treatment of inflammatory diseases.

## Data availability statement

The datasets presented in this study can be found in online repositories. The name of the repository and accession number can be found below: NCBI Gene Expression Omnibus; GSE202260.

## Ethics statement

The studies involving human participants were reviewed and approved by Ethical Review Committees of Yangzhou University. The patients/participants provided their written informed consent to participate in this study. The animal study was reviewed and approved by Institutional Animal Care and Use Committee of Yangzhou University, Yangzhou, China (Approval ID: SYXK [Su] 2017-0044).

## Author contributions

WG and ZL: the conception and design of the study. ZL, XX, MG, and QC: acquisition of data, analysis and interpretation of data. ZL: wrote the main manuscript text, prepared figures, statistical analysis. GL, XJ, WX, and JZ: methodology; WG and DY: Supervision. All authors contributed to the article and approved the submitted version.

## Acknowledgments

This work was supported by the National Natural Science Foundation of China (Grant numbers: 82102901, 81873866 and 81873867), the Natural Science Foundation of Jiangsu Province, China (Grant number: BK20180925); the “Six peaks” Talent Project of Jiangsu Province.

## Conflict of interest

The authors declare that the research was conducted in the absence of any commercial or financial relationships that could be construed as a potential conflict of interest.

## Publisher’s note

All claims expressed in this article are solely those of the authors and do not necessarily represent those of their affiliated organizations, or those of the publisher, the editors and the reviewers. Any product that may be evaluated in this article, or claim that may be made by its manufacturer, is not guaranteed or endorsed by the publisher.
