# Supplementary material for: microRNA-144/451 decreases dendritic cell bioactivity via targeting interferon-regulatory factor 5 to limit DSS-induced colitis
Source: Front Immunol. 2022 Jul 29;13:928593. doi: 10.3389/fimmu.2022.928593 (PMC9372465; doi:10.3389/fimmu.2022.928593)
Supplement: Supplementary file 1 [file DataSheet_1.pdf]

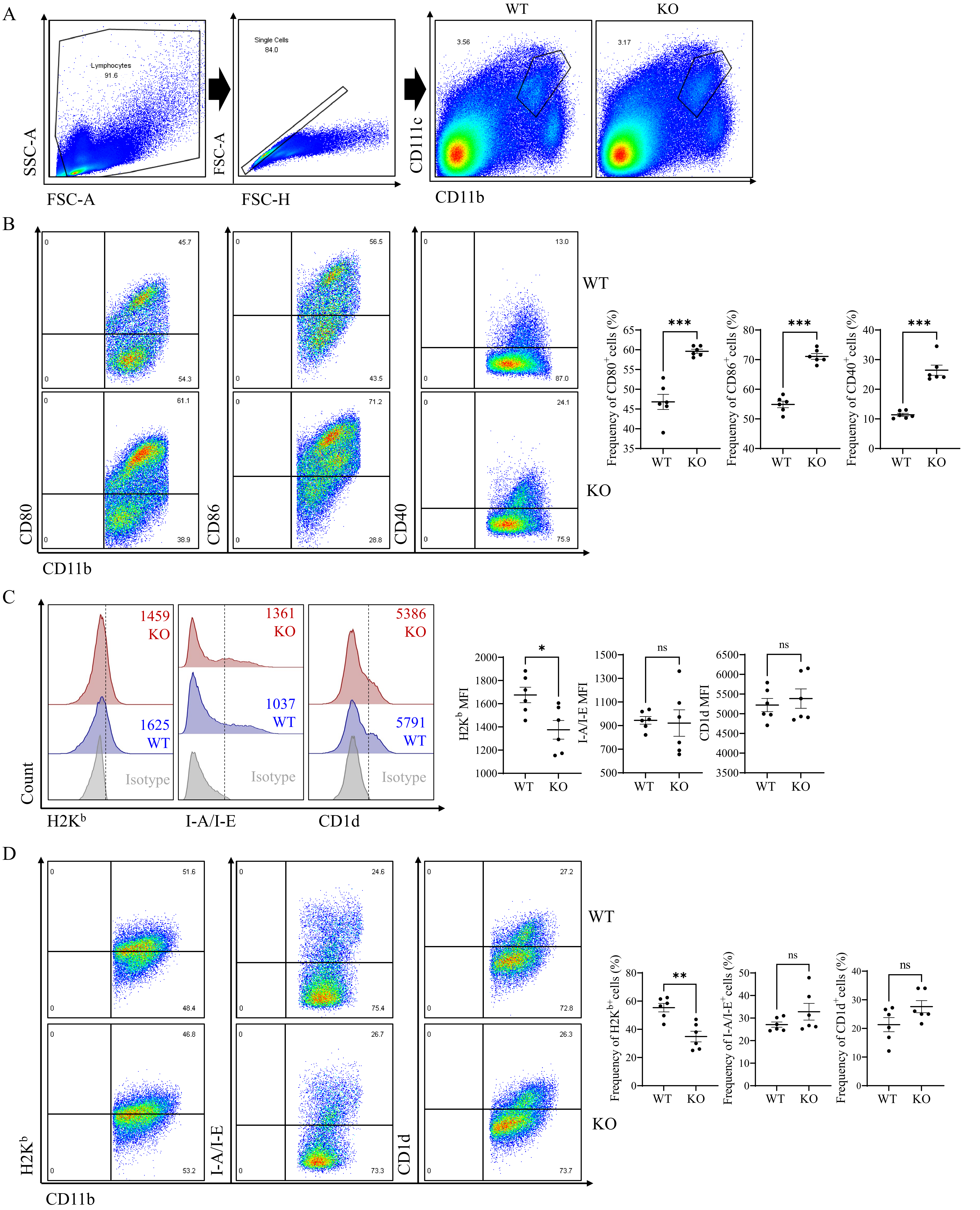
A
WT
KO
Lymphocytes
91.6
Single Cells
84.0
3.56
3.17
SSC-A
FSC-A
FSC-A
FSC-H
CD111c
CD11b
B
45.7
54.3
61.1
38.9
56.5
43.5
71.2
28.8
13.0
87.0
24.1
75.9
WT
KO
CD80
CD86
CD40
CD11b
Frequency of CD80+cells (%)
Frequency of CD86+cells (%)
Frequency of CD40+cells (%)
***
WT
KO
C
1459
KO
1625
WT
Isotype
1361
KO
1037
WT
Isotype
5386
KO
5791
WT
Isotype
Count
H2Kb
I-A/I-E
CD1d
H2Kb MFI
I-A/I-E MFI
CD1d MFI
*
ns
D
51.6
48.4
46.8
53.2
24.6
75.4
26.7
73.3
27.2
72.8
26.3
73.7
WT
KO
H2Kb
I-A/I-E
CD1d
CD11b
Frequency of H2Kb+cells (%)
Frequency of I-A/I-E+cells (%)
Frequency of CD1d+cells (%)
**
ns

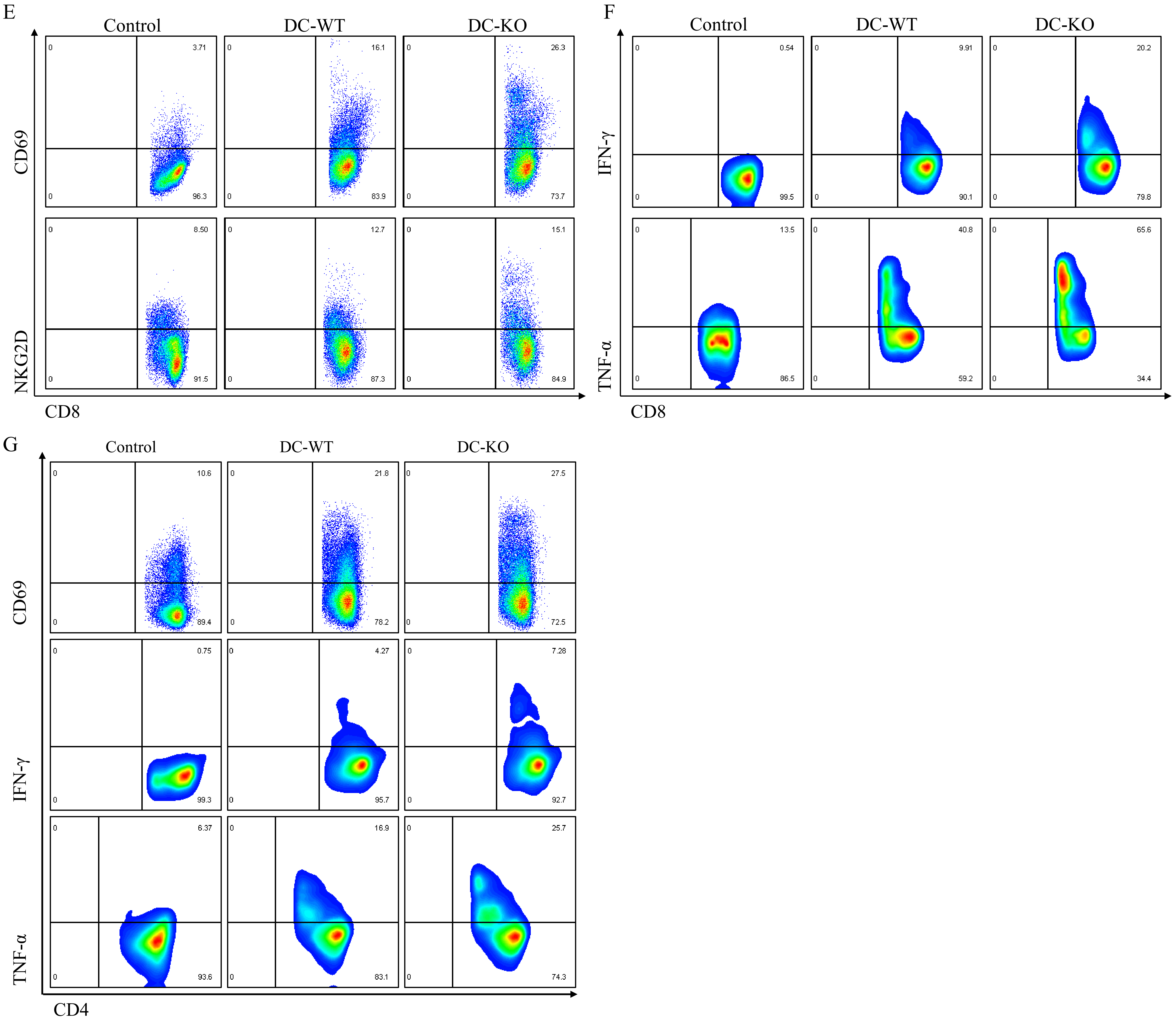

Supplementary Figure 1. miR-144/451 ablation upregulates DCs activation. Frequency of CD11b$^+$CD11c$^+$ DCs in the spleen of WT or miR-144/451 KO mice (A, dot pattern). Expression of CD80, CD86 and CD40 (B, dot pattern), H2Kb, I-A/I-E and CD1d (C, overlay chart and D, dot pattern) on splenic DCs. CD8$^+$T cells (E, F, dot pattern) and CD4$^+$T cells (G, dot pattern) were co-cultured with WT or miR-144/451 KO DCs for 24 h, expression of surface markers CD69, NKG2D and intracellular cytokines IFN-γ, TNF-α were detected. Ns. no significance, *$p<0.05$, **$p<0.01$, ***$p<0.001$.

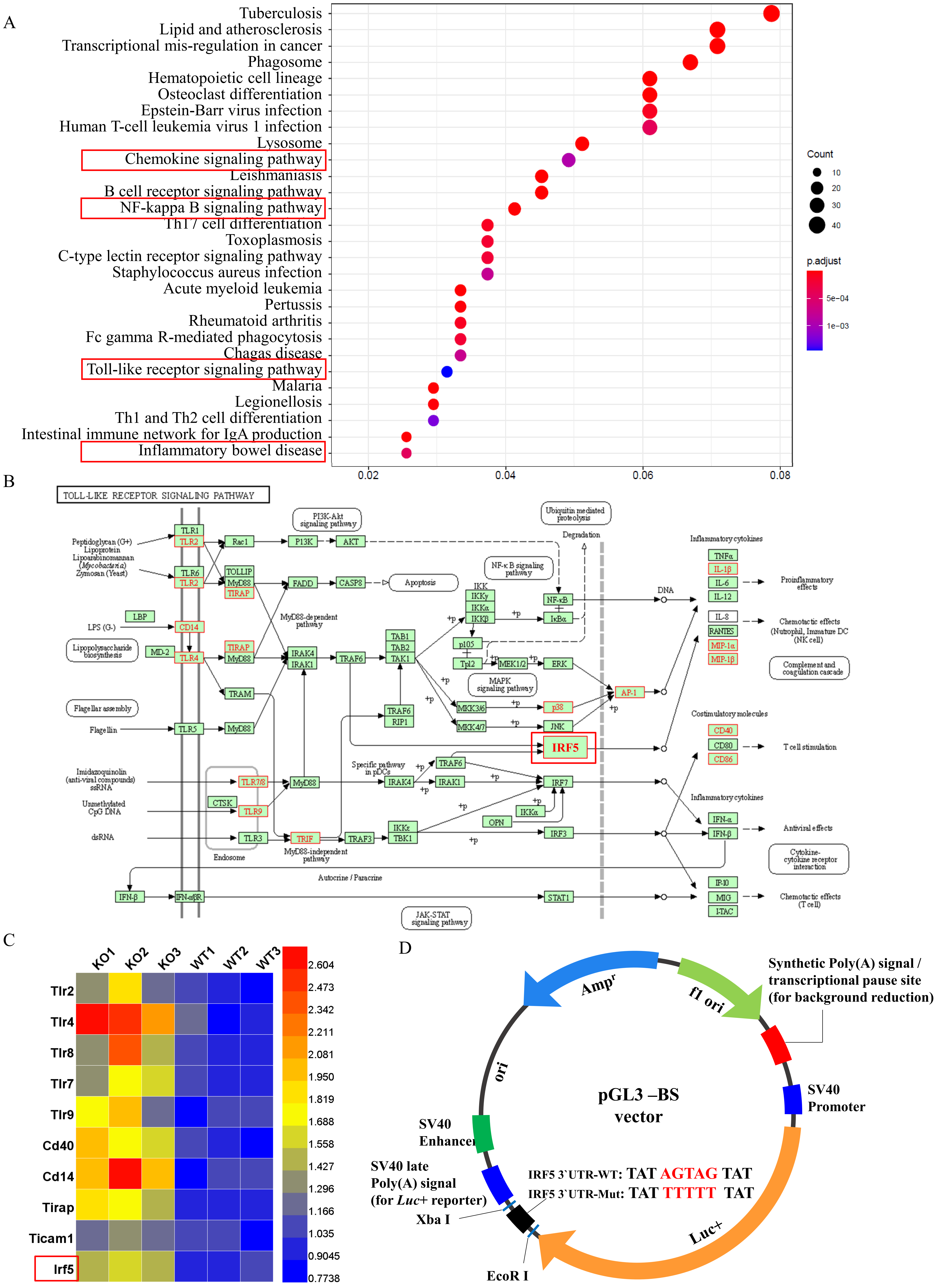

Supplementary Figure 2. Identification of IRF5 as a target of miR-144/451. Differential genes obtained from transcriptome sequencing were analyzed by KEGG cluster profile (A, unregulated genes in KO *vs.* WT, bubble chart). KEGG pathway maps for toll-like receptor signaling pathway (B, mmu04620). Heat map of differential genes in toll-like receptor signaling pathway (C, KO *vs.* WT). Construction of pGL3-IRF5 vector. Native and mutant version 3′-UTRs of IRF5 mRNA- were inserted into the pGL3 vector (D).

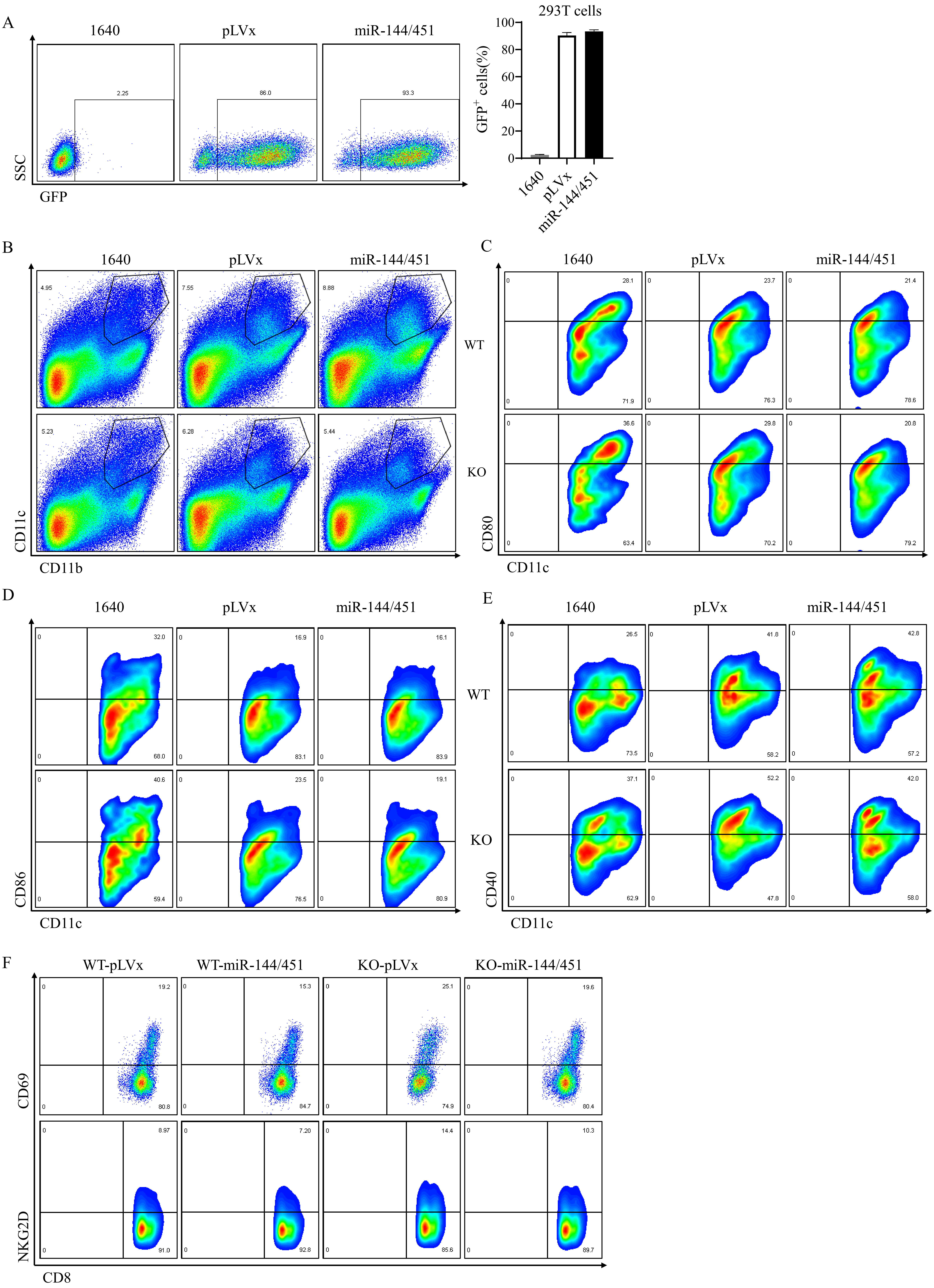
A
1640
pLVx
miR-144/451
2.25
86.0
93.3
SSC
GFP
293T cells
GFP+ cells(%)
100
80
60
40
20
0
1640
pLVx
miR-144/451
B
1640
pLVx
miR-144/451
4.95
7.55
8.88
5.23
6.28
5.44
CD11c
CD11b
C
1640
pLVx
miR-144/451
WT
KO
CD80
CD11c
D
1640
pLVx
miR-144/451
CD86
CD11c
E
1640
pLVx
miR-144/451
WT
KO
CD40
CD11c
F
WT-pLVx
WT-miR-144/451
KO-pLVx
KO-miR-144/451
CD69
NKG2D
CD8

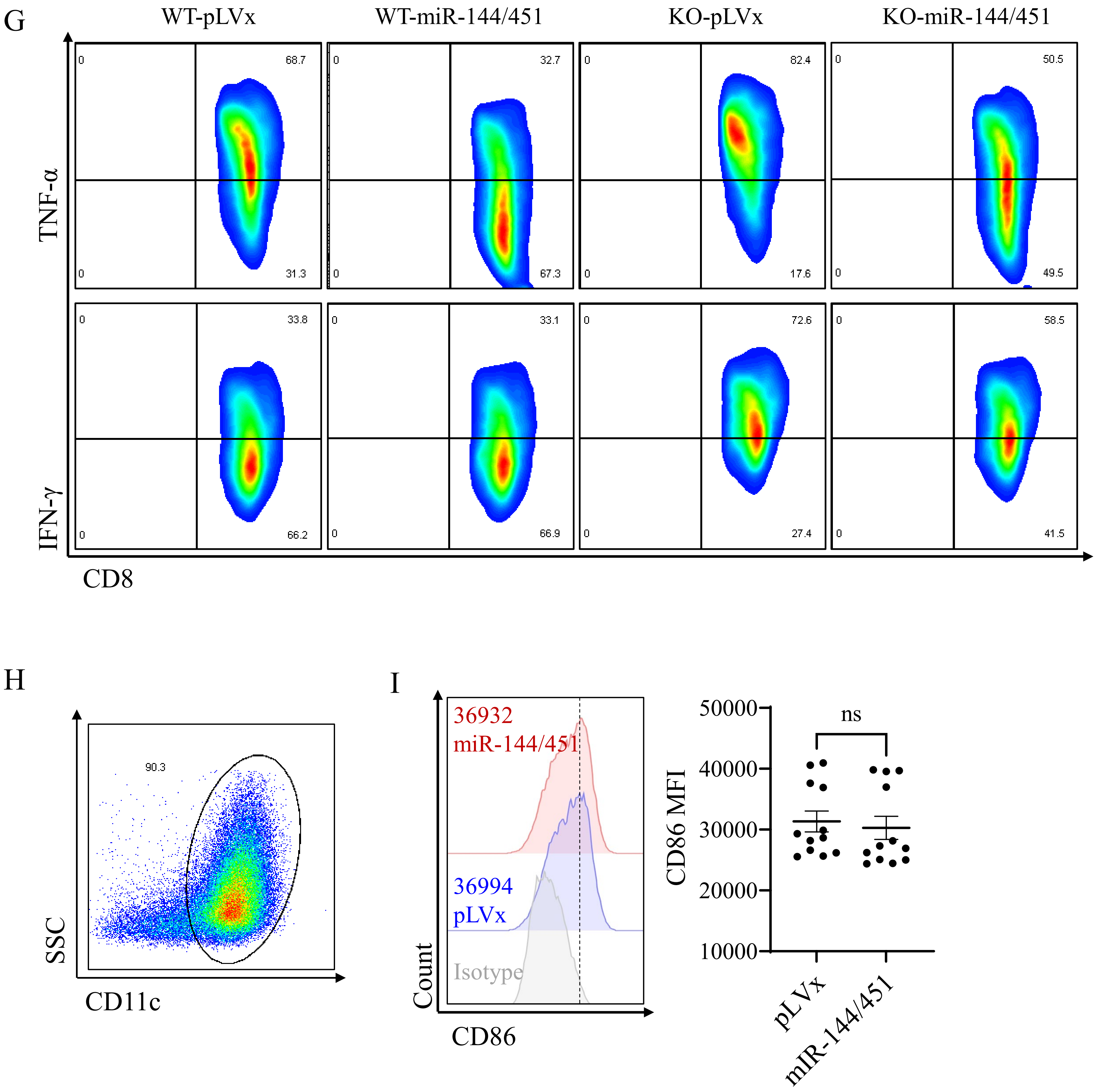

Supplementary Figure 3. Overexpression of miR-144/451 downregulates DCs function. 293T cells infected with lentiviruses (pLVx mock control or miR-144/451) 24 h, frequency of GPF positive cells were detected by FCM (A). Mice splenic DCs were infected with lentiviruses for 24 h, the frequency of DCs (B, contour map) and expression of CD80 (C, contour map), CD86 (D, dot pattern), CD40 (E, contour map) on DCs were detected. $CD8^{+}$T cells were co-cultured with lentiviruses infected DCs for 24 h, surface markers CD69, NKG2D (F, dot pattern) and intracellular cytokines IFN-γ, TNF-α (G, contour map) were detected in $CD8^{+}$T cells. Induction of $CD11c^{+}$ DCs was verified by FCM (H). Expression of CD86 (I) by monocyte-derived DCs was detected after 48 h lentiviruses infection. Ns. no significance.

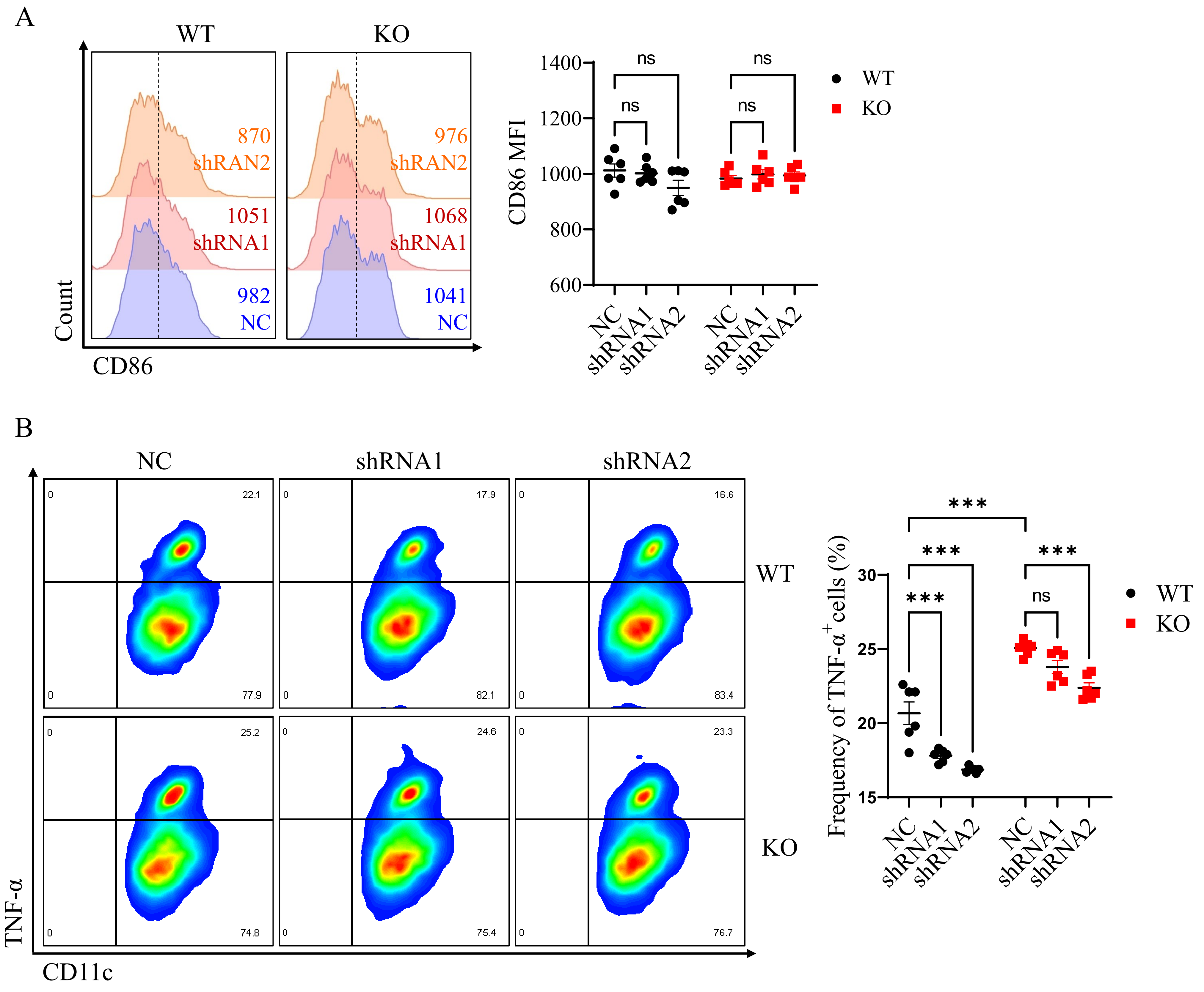

Supplementary Figure 4. Knockdown of IRF-5 inhibits activities of DCs. Expression of CD86 (A, overlay chart) and TNF-α (B, contour map) on splenic DCs after lentiviruses infection. Statistic graph represents the mean fluorescence intensity or frequency of positive cells gated on $CD11b^{+}CD11c^{+}$ cells. Ns. no significance, ***p<0.001.

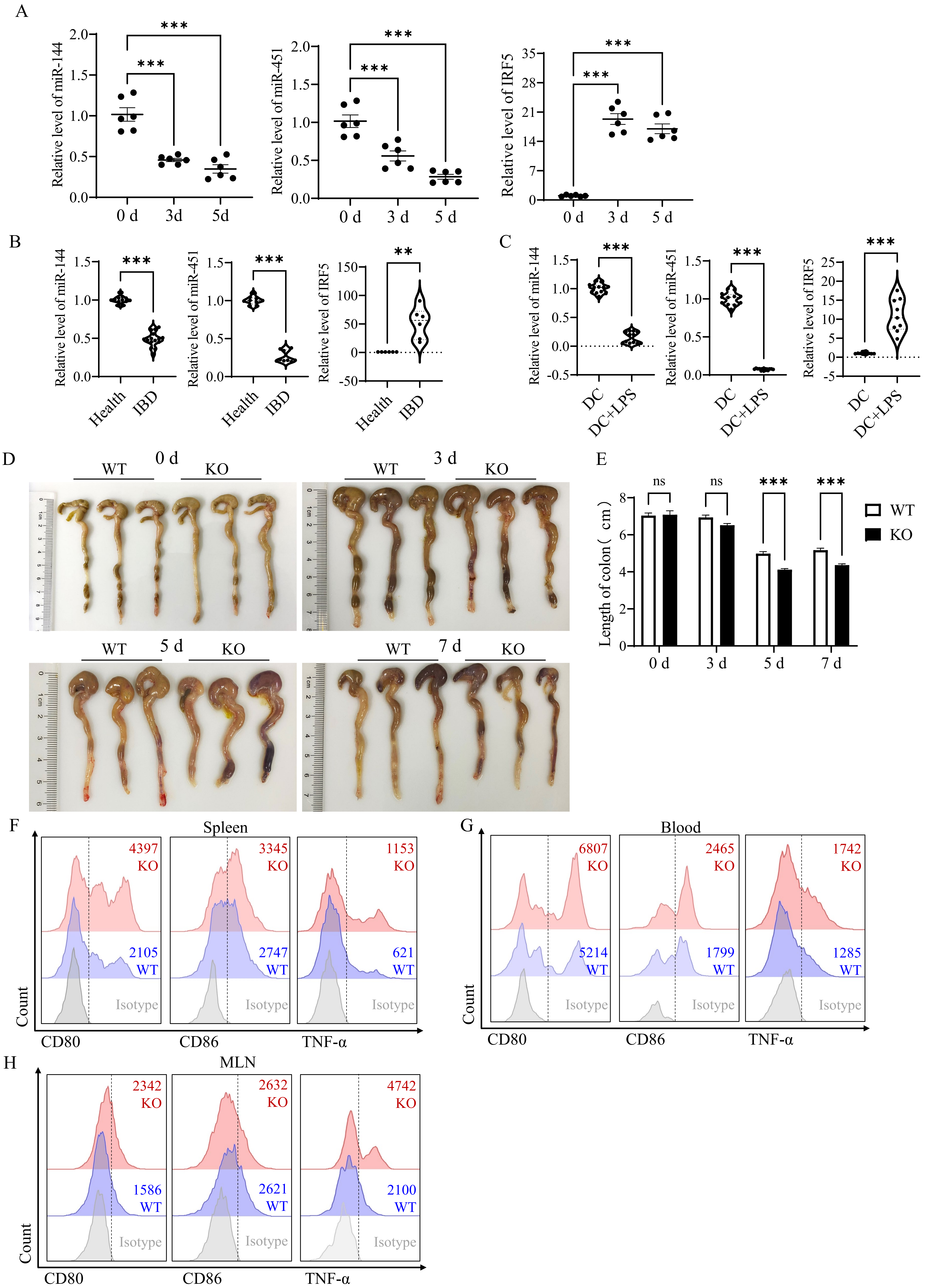

Supplementary Figure 5. miR-144/451 KO exacerbates DSS-induced mice colitis. Expression of miR-144, miR-451 and IRF5 in DCs from colitis mice (A), and IBD patients (B). Expression of miR-144, miR-451 and IRF5 in LPS treated human DCs (C). Colons morphology of colitis mice at day 0, 3, 5 and 7(D), bars represent the length of colons (E). Expression of CD80, CD86 and TNF-α on DCs from spleen (F, overlay chart), Blood (G, overlay chart) and MLN (H, overlay chart) of colitis mice. Ns. no significance, **p<0.01, ***p<0.001.

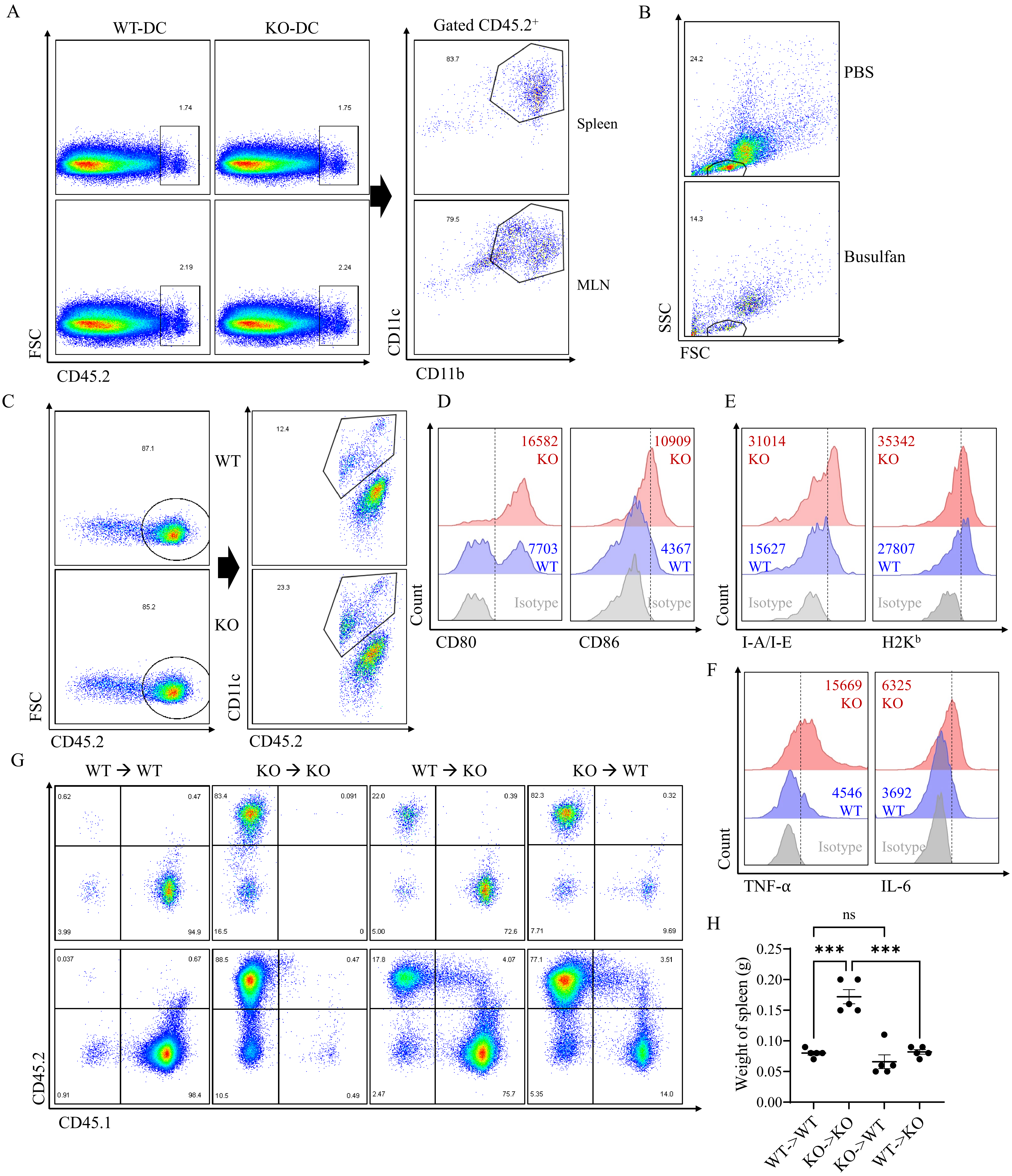

Supplementary Figure 6. DCs or BM transplantation exacerbates DSS-induced mice colitis. CD45.1 recipient mice were treated with 2.5% DSS, and adoptively transferred with WT (CD45.2) or miR-144/451 KO (CD45.2) DCs via tail vein. CD45.2 positive cells and donor DCs were detected by FCM (A). The frequency of PBMCs was checked in busulfan-treated mice (B). Frequency of CD45.2^{+} DCs (C, dot pattern) and expression of CD80, CD86 (D, overlay chart), I-A/I-E, H2Kb (E, overlay chart), TNF-a and IL-6 (F, overlay chart) on CD45.2^{+} DCs were assayed in the spleen of busulfan treated CD45.1 mice. Ratio of CD45.1^{+} and CD45.2^{+} cells was evaluated in bone marrow transplanted mice (G, up: peripheral blood, down: spleen). Spleen weight of bone marrow transplanted mice (H). Ns. no significance, ***p<0.001.

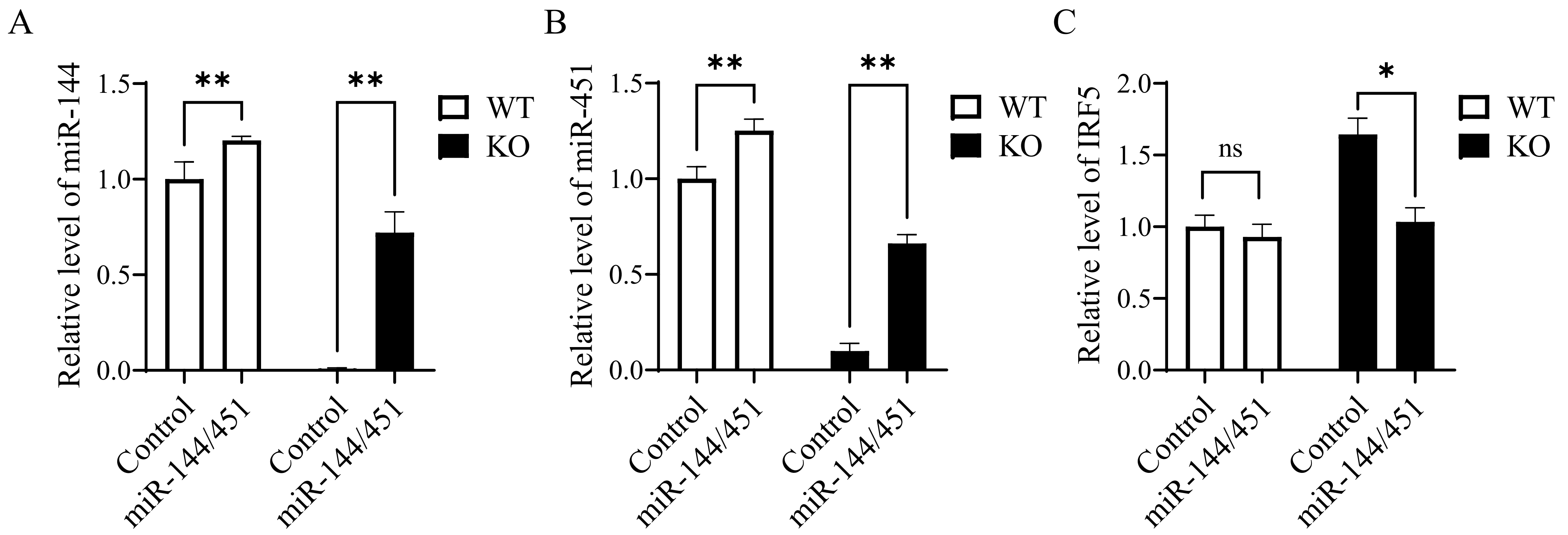

Supplementary Figure 7. Nanoparticles delivery of miR-144/451 ameliorates DSS-induced colitis in KO mice. WT and miR-144/451 KO mice were intraperitoneally injected with chitosan-plasmid nanoparticles daily. Expression of miR-144 (A), miR-451(B) and IRF-5 mRNA(C) was detected in DCs. Ns. no significance, *p<0.05, **p<0.01.
